# Technologiebewertung digitaler Gesundheitsanwendungen für Refundierungsentscheidungen

**DOI:** 10.1007/s10354-021-00881-3

**Published:** 2021-09-16

**Authors:** Reinhard Jeindl, Claudia Wild

**Affiliations:** Austrian Institute for Health Technology Assessment GmbH (AIHTA), Garnisongasse 7/20, 1090 Wien, Österreich

**Keywords:** Digitale Gesundheitsanwendungen, Assessment Frameworks, Refundierung, mHealth, Digitale Gesundheit, Digital Health Applications, Assessment Frameworks, Reimbursement, MHealth, Digital Health

## Abstract

**Hintergrund:**

Für die meisten digitalen Gesundheitsanwendungen (DiGA) liegt wenig Evidenz zum Nutzen vor. Bisher verfügbare Bewertungsinstrumente umfassen häufig nicht alle Domänen eines vollen Health Technology Assessments (HTA). Die Evaluation von DiGA erfordert zusätzlich technologiespezifische Aspekte. Ziel dieser Arbeit war es, verfügbare Bewertungsinstrumente zu analysieren und daraus einen Bewertungsprozess zu konzipieren.

**Methodik:**

Durch eine systematische Literatursuche wurden 6 Bewertungsinstrumente für DiGA ausgewählt und analysiert. Es wurde eine Handsuche zur Beschreibung der Strategien einzelner Länder im Umgang mit DiGA durchgeführt.

**Ergebnisse:**

Studiendesigns wurden in 4 der analysierten Bewertungsinstrumente beschrieben. Eine Risikoklassifikation wurde in 1 Bewertungsinstrument vorgeschlagen. Aspekte der künstlichen Intelligenz wurden in 1 Bewertungsinstrument erhoben. Einzelne Länder weisen unterschiedliche Strategien zur Refundierung von DiGA auf.

**Schlussfolgerungen:**

Die Bewertungsinstrumente für DiGA zeigen eine große Heterogenität. Rezente Entwicklungen verschiedener Länder zeigen Bestrebungen, Regelungen auf nationaler Ebene zu finden. Für einen Bewertungsprozess von DiGA empfiehlt sich ein abgestuftes Vorgehen unter Berücksichtigung von Risikoklassen mit anschließender Bewertung relevanter HTA-Aspekte.

## Abstract (en)

**Background:**

For most digital health applications (DiGA) only limited evidence of benefit is available. Currently available assessment frameworks do not cover all domains of a full health technology assessment (HTA). Additionally, technology-specific aspects are required for the evaluation of DiGA. This work aimed to analyze the available assessment frameworks and design an evaluation process for DiGA.

**Methods:**

By a systematic literature search six assessment frameworks for DiGA were selected and analyzed. A hand search for strategies on DiGA of selected countries was conducted.

**Results:**

Of the analyzed assessment frameworks four described study designs. One assessment framework proposed a risk classification of DiGA. Aspects of artificial intelligence were assessed by one assessment framework. The analyzed countries have differing strategies for reimbursement of DiGA.

**Conclusion:**

Assessment frameworks for DiGA are very heterogeneous. There are efforts to find regulations for DiGA on a national level. When evaluating DiGA, a staged approach considering risk classes with subsequent evaluation of relevant HTA aspects is recommended.

## Einleitung

Digitale Technologien sind Teil unseres Alltags. Dabei hat in den letzten Jahren insbesondere die Nutzung mobiler digitaler Technologien an Bedeutung gewonnen und macht inzwischen den Großteil der Nutzung digitaler Technologien aus [1]. Diese vermehrte Nutzung digitaler Technologien betrifft auch den Gesundheitsbereich. Das Angebot reicht von rein informativen Anwendungen über Anwendungen mit diagnostischen Funktionen sowie Anwendungen für therapeutische Zwecke.

Mit dem Einsatz von digitalen Gesundheitsanwendungen (DiGA) sind große Erwartungen verbunden: DiGA haben das Potenzial, das Monitoring und das Management von Krankheiten zu verbessern, und könnten unnötige Wege für Patient*innen und Therapeut*innen vermeiden. Durch eine Fernüberwachung, insbesondere bei chronischen Erkrankungen, könnten Krankenhausaufenthalte vermieden werden. Bei kontinuierlich gemessenen Vitalparametern könnte eine Entgleisung eines Werts rasch erkannt und entsprechende Maßnahmen könnten gesetzt werden. Für die Mehrzahl der verfügbaren DiGA liegt bislang aber wenig Evidenz zum tatsächlichen Nutzen vor. Die gründliche Evaluation einer DiGA erfordert zusätzlich zu einer Nutzen-Risiko-Abwägung auch technologiespezifische Aspekte. Bisher verfügbare Bewertungsinstrumente umfassen häufig nicht alle Domänen eines vollen Health Technology Assessments (HTA). Entscheidungsträger*innen werden somit vor neue Herausforderungen in der Evaluation dieser Applikationen gestellt.

Durch die neue EU-Verordnung 2017/745 über Medizinprodukte, die im Mai 2021 in Kraft tritt, ist auch medizinische Software als Medizinprodukt einzustufen, wenn die Software laut Hersteller dazu bestimmt ist, medizinische Zwecke zu erfüllen [2]. Zusätzlich zu Medizinproduktsoftware („medical device software“, etwa die Software eines EKG-Gerätes) kann auch Software als Medizinprodukt („software as a medical device“, etwa eine DiGA mit diagnostischen und/oder therapeutischen Funktionen) betrachtet werden. Durch diese neue EU-Verordnung 2017/745 wird es zu einer Höherstufung der Risikoklasse vieler digitaler Gesundheitsanwendungen kommen [3].

Erste DiGA werden bereits als medizinische Leistung von einzelnen Krankenkassen in Deutschland, den Niederlanden und Belgien in Selektivverträgen übernommen. Für eine verbesserte Regulierung von medizinischer Software wird eine generelle, nationale Refundierung von geeigneten DiGA empfohlen [4, 5]. In Europa hat man in einzelnen Ländern begonnen, einen Prozess der Registrierung aufzusetzen, der infolge für einzelne DiGA auch zu einer Refundierung durch Sozial- und Krankenversicherungen führen kann.

Die Intention dieser Arbeit, die auf einem ausführlichen Projektbericht basiert [6], war es, eine kritische Analyse verschiedener Bewertungsinstrumente für DiGA durchzuführen und eine Übersicht unterschiedlicher Zugänge einzelner europäischer Länder bei der Einführung und Implementierung von DiGA im Gesundheitswesen aufzuzeigen. Daraus abgeleitet, verfolgte die Arbeit das Ziel, eine Orientierungshilfe über den Umgang mit DiGA für Entscheidungsträger*innen zu erstellen.

## Methodik

### Suche nach Bewertungsinstrumenten:

In einem ersten Schritt wurde eine systematische Literatursuche (im Juni 2020) in den Datenbanken Medline, Embase, Cochrane und der HTA Datenbank (Centre for Reviews and Dissemination) nach Bewertungsinstrumenten für DiGA durchgeführt und durch eine zusätzliche Handsuche ergänzt. Die systematische Suche diente einer Aktualisierung des systematischen Reviews von Vis et al. [7] zu Bewertungsinstrumenten für DiGA. Bewertungsmodelle von DiGA, die in englischer oder deutscher Sprache ab März 2018 publiziert wurden, wurden inkludiert.

Um weitere Bewertungsinstrumente für DiGA zu identifizieren, wurde ebenfalls im Juni 2020 eine Anfrage an alle Mitglieder des International Network of Agencies for Health Technology Assessment (INAHTA) gestellt. Dabei wurde erhoben, welche Bewertungsinstrumente von verschiedenen HTA-Institutionen als adäquat für DiGA erachtet werden und ob die jeweilige HTA-Institution an einer Entwicklung oder Adaptierung eines Bewertungsinstruments für DiGA arbeitet.

### Auswahl der Bewertungsinstrumente und Datenextraktion:

Aus der systematischen Suche und den Antworten der INAHTA Befragung wurden jene Bewertungsinstrumente für eine genauere Analyse ausgewählt, die aktuell (ab März 2018 publiziert), ausgereift (fortgeschritten in der Entwicklung), und für welche eine Anwendbarkeit (an konkreten DiGA) zu erwarten war. Bereits etablierte Bewertungsinstrumente wurden nicht gewählt, wenn diese zwar für den Bereich eHealth aufgestellt sind, jedoch die Entwicklungen im Bereich mobiler DiGA („mHealth“) nicht ausreichend berücksichtigten.

Die ausgewählten Bewertungsinstrumente wurden miteinander verglichen und auf Ähnlichkeiten und Unterschiede analysiert. Kriterien für die Datenextraktion aus den Bewertungsinstrumenten waren Institution/Land, Finanzierung, Zeitpunkt der Veröffentlichung, Zielgruppe, Entwicklungsstatus, Anwendungsbereich, Berücksichtigung von Risikoklassen, Evidenzerfordernisse, Zeitpunkt der Bewertung, Format, Bewertungsdomänen, technologiespezifische Aspekte, Involvierung Patient*innenvertretung und sonstige Spezifika des Bewertungsinstruments.

### Auswahl Länderstrategien und Analysekriterien:

Für die Analyse von Länderstrategien wurde eine Auswahl an Ländern mit ähnlichem Gesundheitssystem (extramurale Versorgung durch niedergelassene Ärzt*innen, deren Leistungserbringung über Verträge mit Sozialversicherungen und Krankenkassen abgegolten werden) getroffen: Deutschland, Belgien Niederlande, Frankreich. Die Auswahl der Länder basierte zusätzlich auf dem MTRC-Bericht „Reimbursement Landscape for Health Apps in Europe“ [8] und wurde durch eine Handsuche auf den Websites der entsprechenden nationalen Institutionen ergänzt. Die regulatorischen und organisatorischen Herangehensweisen der Länder wurden als Analysekriterien herangezogen.

### Konzeption einer Assessment-Orientierungshilfe für Österreich:

Basierend auf der Analyse und den jeweiligen Limitationen der einzelnen Bewertungsinstrumente sowie der Länderanalysen wurde zur Konzeption einer Assessment-Orientierungshilfe für Österreich ein abgestuftes Vorgehen mit Kombination von Bewertungsinstrumenten erstellt.

## Ergebnisse

### DiGA-Bewertungsinstrumente

Insgesamt wurden durch die systematische Literatursuche, die Befragung der INAHTA-Mitglieder sowie die in Vis et al. genannten [7] Bewertungsinstrumente für DiGA 14 Bewertungsinstrumente identifiziert [9–22]. Aus diesen wurden anhand der genannten Kriterien 6 Bewertungsinstrumente [9–14] für eine detaillierte Analyse ausgewählt, welche in Tab. 1 dargestellt sind. Die Bewertungsinstrumente wurden von Universitäten und nationalen Institutionen entwickelt.

**Table 1:** 

	*HTA Module for Mobile Medical Applications *[9], AHTA (Adelaide Health Technology Assessment University of Adelaide), Australia	*Evidence Standards Framework for Digital Health Technologies *[10], NICE (National Institute for Health and Care Excellence), United Kingdom	*Digital Health Care Services/Digi-HTA* [11], Faculty of Medicine, University of Oulu, Finland	*Design & Evaluation Framework for Digital Interventions/DEDHI* [12], Center for Digital Health Interventions, University of St. Gallen & ETH Zürich, Switzerland	*Digital Health Scorecard* [13], Armstrong Institute for Patient Safety and Quality & Johns Hopkins Medicine, Baltimore, MD, USA	*mHealth Agile Development & Lifecycle *[14], Faculty of Medicine, University of Ottawa, Canada
*Finanzierung*	Australian Government Research Training Program Scholarship and University of Adelaide, School of Public Health	National Health Service England	Regional Council of Northern Ostrobothnia (funding from the European Regional Development Fund), Funding by the Northern Ostrobothnia Hospital District	Health Promotion Switzerland, and the European Social Fund and the Free State of Saxony (Grant no. 100310385)	Das Framework entstand ohne Finanzierung aus dem öffentlichen, kommerziellen oder gemeinnützigen Sektor	Public Health Agency of Canada, Sanofi-Pasteur, the Canadian Institutes of Health Research, the Ottawa Hospital and Health Canada
*Jahr*	April 2020	März 2019	November 2019	November 2019	Mai 2019	September 2018
*Zielgruppe*	HTA Expert*innen, Entscheidungsträger*Innen	Technologieentwickler*Innen, Entscheidungsträger*Innen	HTA Expert*innen, Entscheidungsträger*Innen	Wissenschafter*Innen, Evaluator*Innen	Unabhängige Evaluator*Innen	Technologieentwickler*Innen, Entscheidungsträger*Innen
*Status Framework*	In Entwicklung	Etabliert, in Erprobung	In Entwicklung/in Erprobung	In Erprobung	In Entwicklung	In Erprobung
*Anwendungsbereich/Titel*	Mobile Medical Application (MMA)	Digital Health Technologies (DHT)	mHealth, Künstliche Intelligenz, Robotik	Digital Health Interventions (DHI)	Digital Health Products	mHealth/Digital Health Products
*Risikoklassen*	Keine Erwähnung von Risikoklassen	Risikoklassifikation (1, 2, 3a/3b) entsprechend der Funktionalität der DHT	Keine definierten Risikoklassen, aber deskriptive Fragen nach klinischer Konsequenz	Keine Erwähnung von Risikoklassen	Keine definierten Risikoklassen, aber Differenzierung nach Funktionalitäten und deren klinischen Konsequenz	Keine definierten Risikoklassen, aber Differenzierung zwischen Niedrigrisiko- (keinerlei Risiken für Anwender*Innen) und Hochrisikoanwendungen
*Evidenzerfordernisse*	Keine Erwähnung von Studiendesigns, nur von Vergleichsinterventionen, Analyse von Schaden durch Fehlinformation, ethische Aspekte	Evidenzerfordernisse entsprechend der Risikoklasse (RK), je RK Minimalerfordernisse vs. Best Practice, z. B. 3a: Beobachtungsstudie vs. Interventionsstudie 3b: Interventionsstudien/RCT Komparatoren, relevante Endpunkte	Empfohlene Studiendesigns: Fallstudien, RCTs, systematische Reviews	Orientierung am MOST Framework zu Evidenzerfordernissen in Produktentwicklungsphasen Phase 1: Studien zu Durchführbarkeit Phase 2: Studien zu Optimierung Phase 3: RCTs Phase 4: kontinuierliche Erhebungen zu Reichweite, Impakt und Nebenwirkungen	Keine Erwähnung von Studiendesigns, nur kritische Bewertung der Evidenz bezüglich Impakt auf definierte klinische Endpunkte. Vergleiche zu Goldstandard	Orientierung am IDEAS Framework zu Evidenzerfordernissen in Produktentwicklungsstadien Phase 2: Kohortenstudien, Fallserien Phase 3: RCTs (sofern mHealth-Produkte als Medizinprodukte definiert sind), A/B testing (Testung von App-Versionen)
*Zeitpunkt der Anwendung des Frameworks/Intention*	Vor Refundierung	Vor Refundierung, zur Evidenzgenerierung	Vor Refundierung , zur Evidenzgenerierung, Post-market-Surveillance	Zur Evidenzgenerierung, Post-market-Surveillance	Zur Evidenzgenerierung, Post-market-Surveillance	Zur Evidenzgenerierung, Post-market-Surveillance
*Format des Frameworks*	Herkömmliche HTA-Checkliste	Fragen zum Kontext zur Einordnung der DHT in eine Risikoklasse, tabellarische Evidenzerfordernisse (kumulativ, aufsteigend)	Fragebogen HTA-Checkliste	Checkliste zu F&E sowie Implementierung von DHIs	Scorecard (Scores sind nicht definiert)	Fragebogen zu F&E von mHealth-Apps im Lebenszyklus nach IDEAS
*Bewertungsdomänen*	Technische Aspekte, Effektivität (Nutzen), Sicherheit, Kosten, Kosteneffektivität, organisatorische, legistische, ethische und soziale Aspekte	1: Plausibilität, Relevanz, Akzeptanz, gleicher Zugang, Genauigkeit 2: Verlässlichkeit, Wert für Zielpopulation, Qualität und Datensicherheit 3a/3b: klinische Effektivität anhand relevanter Endpunkte 1–3: ökonomischer Impakt	Technische Aspekte, Effektivität (Nutzen), Sicherheit, ökonomische Aspekte, Anwenderaspekte und Zugang, organisatorische Aspekte, Interoperabilität	Benutzerfreundlichkeit, inhaltliche Qualität, Privatsphäre und Datenschutz, Verantwortlichkeit, Adhärenz, Ästhetik, empfundener Nutzen, Effektivität (Nutzen), Qualität des Service, Personalisierung, empfundenes Engagement, ethische Aspekte, Sicherheit	4 Domänen: technische Aspekte, klinische Effektivität (Nutzen), Benutzerfreundlichkeit, Kosten	Phase 1 der Entwicklung: technische Aspekte, Performanz und Datensicherheit Phase 2: Anwenderaspekte, Akzeptanz Phase 3: Nutzen
*Technologiespezifische Aspekte:* *Datenschutz* *Datensicherheit* *Umgang mit Updates* *Künstliche Intelligenz (KI)*	Datenschutz: ja Datensicherheit: ja Umgang mit Updates: ja Künstliche Intelligenz: k. A.	Datenschutz: k. A Datensicherheit: k. A Umgang mit Updates: k. A Künstliche Intelligenz: nur fixe Algorithmen, NICHT adaptive Algorithmen	Datenschutz: ja Datensicherheit: ja Umgang mit Updates: ja Künstliche Intelligenz: ja	Datenschutz: ja Datensicherheit: ja Umgang mit Updates: ja Künstliche Intelligenz: nein	Datenschutz: ja Datensicherheit: ja Umgang mit Updates: nein Künstliche Intelligenz: nein	Datenschutz: ja Datensicherheit: ja Umgang mit Updates: ja Künstliche Intelligenz: nein
*Involvierung* Patient*Innenvertretung	k. A.	k. A.	k. A.	Nein	Nein	k. A.
**Framework-Spezifika** **Resümee**	Systematischer Review zu derzeit verwendeten Evaluations-Frameworks. Derzeit kein eigenständiges Framework, nur Zusammenschau. Traditionelle HTA-Perspektive	Stellt die geforderte Evidenz in Relation zum möglichen Risiko der DHT. Auflistung geforderter Studiendesigns je nach Risikoklasse Einziges hoch entwickeltes Framework zur sofortigen Anwendung	Framework basiert auf systematischem Review, Interviews und Workshop. Vereint mHealth, KI und Robotik in einem Framework Ethische, soziale und rechtliche Aspekte sind explizit kein Bestandteil dieses Frameworks	Framework behandelt unterschiedliche Entwicklungsphasen einer Digital-Health-Intervention, die Implementierungsphase wird detailliert berücksichtigt	Die Digital Health Scorecard soll jeweils einmal vor als auch nach Marktzulassung angewendet werden	Das Framework vereint einen „agilen“ Entwicklungsprozess aus Perspektive von Technologienentwickler*Innen mit Methoden zur Nutzenbewertung

In den Antworten der Mitglieder von INAHTA zeigte sich, dass sich viele HTA-Institutionen am National Institute for Health and Care Excellence (NICE) Evidence Standards-Bewertungsinstrument [10] (vgl. Tab. 1) orientieren und dessen Nutzung für eine erste Evaluierung von DiGA in den jeweiligen Ländern (Healthcare Improvement Scotland [HIS, Schottland], Health Technology Wales [HTW, England], Agency of Health Quality and Assessment of Catalonia [AquAS, Spanien], Canadian Agency for Drugs and Technologies in Health [CADTH, Kanada] und das Institut für Qualität und Wirtschaftlichkeit im Gesundheitswesen [IQWiG, Deutschland]) geplant ist. Ein weiteres Bewertungsinstrument wurde von Adelaide Health Technology Assessment (AHTA, Australien) entwickelt, das bisher noch keine Anwendung fand – das HTA Module for Mobile Medical Applications [9] (vgl. Tab. 1). Interesse, dieses Bewertungsinstrument zu nutzen, wurde von der Health Technology Assessment Unit (UVT, Italien) bekundet. Weitere Bewertungsinstrumente für DiGA werden derzeit von AquAS (Spanien) und dem Institut National d’Excellence en Santé et en Services Sociaux (INESSS, Kanada) entwickelt.

Die ausgewählten Bewertungsinstrumente können für DiGA aller medizinischen Fachgebiete und Populationen eingesetzt werden (im Gegensatz zu Bewertungsinstrumenten, die sich speziell einem Fachgebiet, einer Indikation oder Population widmen). Die Bewertungsinstrumente bedienen sich unterschiedlicher Bezeichnungen („Mobile Medical Applications“, „Digital Health Technologies“, „Digital Health Interventions“ und „Digital Health Products“) und unterscheiden sich nach Format (Checkliste, Fragebogen, Tabelle), Spektrum der Bewertungsdomänen, technologiespezifischen Aspekten und Zielgruppe.

Nach Analyse der 6 ausgewählten Bewertungsinstrumente für DiGA zeigte sich, dass 4 von 6 Bewertungsinstrumenten Vorschläge zu Studiendesigns für DiGA nannten [10–12, 14]. Obwohl die Bewertungsinstrumente das Risiko bei Anwendungsfehlern mit klinischer Konsequenz zwar berücksichtigen, schlägt nur 1 Bewertungsinstrument eine präzise Einteilung nach definierten Risikoklassen der zu bewertenden DiGA vor: das NICE Evidence Standards-Bewertungsinstrument [10]. Vom NICE Evidence Standards Framework explizit ausgenommen sind jedoch DiGA, welche auf künstlicher Intelligenz mit adaptiven Algorithmen basieren (selbstlernende Algorithmen, die ihr Verhalten, basierend auf verfügbaren Informationen, kontinuierlich und automatisiert anpassen).

Wesentliche Aspekte der künstlichen Intelligenz wurden hingegen in einem von 6 Bewertungsinstrumenten berücksichtigt, dem sog. „Digi-HTA“ Framework [11]. Rechtliche, soziale und ethische Aspekte werden von diesem Bewertungsinstrument aufgrund der Komplexität und des Zeitaufwands ausgeklammert. Die Themen Datensicherheit und Datenschutz werden von diesem Bewertungsinstrument ebenfalls nicht bewertet, da deren Prüfung ausgelagert und von Datenschutzexpert*innen vorgesehen ist.

Die Evidenzstufen des NICE Evidence Standards Frameworks zeigen Ähnlichkeiten mit den Risikoklassen der EU-Verordnung 2017/745 über Medizinprodukte und sind dennoch unterschiedlich definiert (vgl. Tab. 2).

**Table 2:** 

Risikoklassen nach EU-Verordnung 2017/745 über Medizinprodukte (Regel 11) [2]	Evidenzstufen nach NICE Evidence Standards Framework [10]
*Risikoklasse 1*	*Stufe 1*
Jede Software, die nicht in Risikoklasse 2a oder höher eingeteilt wird	Digitale Gesundheitsanwendungen ohne individuell messbare Effekte auf die Gesundheit von Anwender*innen. Organisatorische Anwendungen auf Systemebene *Beispiele:* elektronische Gesundheitsakte, Krankenhaus-Informations-Systeme
*Risikoklasse 2a*	*Stufe 2*
Software, welche Information zur Entscheidung einer Diagnose oder therapeutischen Zwecken zur Verfügung stellt, ohne jedoch dabei ernstere Gesundheitsschäden verursachen zu können (s. nachfolgende Risikoklassen) Software, die physiologische Messwerte aufzeichnet, solange es sich dabei nicht um Vitalparameter handelt	Digitale Gesundheitsanwendungen, welche Gesundheitsinformationen anbieten, einfache Monitoringfunktionen übernehmen, oder Anwendungen zur Kommunikation *Beispiele:* Anwendungen mit Empfehlungen für einen gesunden Lebensstil, digitaler Kopfschmerzkalender, Anwendungen für Videosprechstunden mit Therapeut*innen
*Risikoklasse 2b*	*Stufe 3a*
Software, welche Information zur Entscheidung einer Diagnose oder therapeutischen Zwecken zur Verfügung stellt, bei der ernstere Gesundheitsschäden oder die Notwendigkeit einer chirurgischen Intervention entstehen können Software, die Vitalparameter aufzeichnet (Messwerte, bei denen eine Änderung eine unmittelbare Gefahr für die Patient*innen darstellen kann)	Digitale Gesundheitsanwendungen, die der Prävention und dem Selbstmanagement von Krankheiten dienen. Digitale Gesundheitsanwendungen mit individuell messbaren Effekten auf die Gesundheit von Anwender*innen. Sie können parallel zu konventioneller Therapie eingesetzt werden *Beispiele:* Anwendungen zur Raucherentwöhnung oder Gewichtsreduktion
*Risikoklasse 3*	*Stufe 3b*
Software, welche Information zur Entscheidung einer Diagnose oder therapeutischen Zwecken zur Verfügung stellt, bei der diese Entscheidung ein Todesereignis oder irreversible Schäden der Gesundheit verursachen kann	Digitale Gesundheitsanwendungen, die der Diagnostik und Therapie von Krankheiten dienen. Digitale Gesundheitsanwendungen mit klinischen Konsequenzen (durch aktives Monitoring oder Berechnungen). Digitale Gesundheitsanwendungen mit individuell messbaren Effekten auf die Gesundheit von Anwender*innen *Beispiele:* Anwendungen, die anhand eingegebener Symptome Diagnosevorschläge machen, Anwendungen, die kognitive Verhaltenstherapie für Angststörungen anbieten, Anwendungen, die mit einem externen Sensor oder Implantat verknüpft sind und dessen Daten automatisch und kontinuierlich für Fernmonitoring gesendet werden

Aspekte verschiedener HTA-Domänen können je nach bewerteter DiGA als besonders relevant betrachtet werden. Für die Domäne Wirksamkeit stellt etwa diagnostische Genauigkeit eines Algorithmus oder die Reduktion von Symptomatik bei therapeutischen DiGA relevante Beurteilungskriterien dar. Für die Domäne Sicherheit gilt es, unerwünschte Nebenwirkungen wie etwa durch Fehlinformation der DiGA zu berücksichtigen. In Bezug auf Kosteneffektivität können durch DiGA Kosten vermieden, aber auch Mehrkosten verursacht werden. Als relevante organisatorische Auswirkungen sind Überdiagnostik und Interoperabilität relevante Bewertungskriterien. Die zu bewertenden Endpunkte richten sich nach Funktionsweise, Einsatzgebiet und Zweckbestimmung der DiGA.

Datenschutz bei DiGA nimmt eine besonders relevante Rolle ein, da bei Datenschutzmängeln personenbezogene Gesundheitsdaten ohne Zustimmung der Nutzer*in weitergegeben werden können. Nicht ausreichende Nachvollziehbarkeit der künstlichen Intelligenz und Intransparenz der verwendeten Algorithmen stellen ethische Problemfelder dar. Fragen zu Haftung bei Fehldiagnosen oder unerwünschten Nebenwirkungen durch DiGA stellen einen weiteren relevanten Aspekt in der Bewertung von DiGA dar.

### Länderstrategien

In Europa hat man in einzelnen Ländern begonnen, regulatorische Maßnahmen zu setzen und Prozesse für eine Registrierung von DiGA aufzusetzen und infolge eine Bewertung einzelner DiGA vorzunehmen, die auch zu einer Refundierung durch Sozial- und Krankenversicherungen führen kann.

#### Belgien:

Der administrative Rahmen für die Regulierung von DiGA bildet die Plattform mHealthBelgium (https://mhealthbelgium.be/), die auf Initiative der belgischen Regierung im Rahmen der eHealth-Strategie entstand und in enger Kooperation mit Herstellern geführt wird. Zentraler Bestandteil des Rahmenwerks ist die sog. „Validierungspyramide“ für Health-Apps. Diese Plattform zentralisiert alle relevanten und erforderlichen Informationen von CE-zertifizieren DiGA für Anwender*innen. Die Validierungspyramide besteht aus 3 Ebenen; eine App tritt immer auf der untersten Ebene (DiGA ist als Medizinprodukt zugelassen, und die Einhaltung der EU-Datenschutzverordnung wurde überprüft) ein und kann in der Hierarchie aufsteigen auf Ebene 2 (Interoperabilität und Konnektivität mit den nationalen eHealth-Anforderungen wurden überprüft, Sicherheitsrisikobewertung liegt vor) und auf Ebene 3 (DiGA kann sozioökonomischen Nutzen nachweisen). Nur DiGA in der obersten Ebene werden für eine Refundierung im Rahmen der (öffentlichen) Krankenversicherungen erwogen. Bislang sind 26 Gesundheitsapplikationen auf der Plattform mHealthBelgium vertreten: 18 auf Stufe 1, 8 auf Stufe 2 und keine auf Stufe 3 der Validierungspyramide.

#### Frankreich:

In Frankreich werden DiGA im Rahmen des bestehenden Regelwerks für Erstattungsentscheidungen zu Medizinprodukten eingegliedert. Vor der Aufnahme in die Liste der erstattungsfähigen Produkte (LPPR/„Liste des Produits et Prestations Remboursables“) werden alle Medizinprodukte von CNEDiMTS, dem Nationalen Komitee zur Evaluierung von Medizinprodukten und Gesundheitstechnologien, auf ihren Zusatznutzen evaluiert und in Klassen von diagnostisch-therapeutischem Nutzen (ASMR 1–4) klassifiziert. Die Bewertungen basieren auf 2 Methodenpapieren (2016 [23] und 2019 [24] veröffentlicht), die Nachweise auf hohem Evidenzniveau einfordern. Bislang ist eine DiGA in die Erstattungsliste aufgenommen worden.

#### Niederlande:

Mit der Einrichtung des National eHealth Living Lab (NeLL, https://nell.eu) im Jahr 2018, einem Netzwerk aus Gesundheitsadministration, Herstellern und akademischen Institutionen, hat das niederländische Gesundheitsministerium die ersten Schritte zu einem einheitlichen nationalen Apps-Archiv unternommen. NeLL hat die Aufgabe, die DiGA einheitlich nach Kriterien (nachgewiesene Wirksamkeit, Anwenderfreundlichkeit und transparente Kommunikation zu Apps) zu bewerten. Derzeit sind zahlreiche Anwendungen auf der NeLL-Plattform, von denen eine die Kennzeichnung „NeLL-kompatibel“ trägt.

#### Deutschland:

In Deutschland wurden mit dem Digitale-Versorgung-Gesetz (DVG, Dez. 2019) das Verschreiben und die Refundierung von ausgewählten DiGA durch Vertragsärzt*innen und -therapeut*innen beschlossen. Voraussetzung für ein Verschreiben und eine Refundierung einer DiGA ist, dass diese im Verzeichnis erstattungsfähiger digitaler Gesundheitsanwendungen (DiGA-Verzeichnis, https://diga.bfarm.de/de) gelistet ist. Dieses DiGA-Verzeichnis wird durch das Bundesinstitut für Arzneimittel und Medizinprodukte (BfArM) verwaltet. Für DiGA mit geringem Risiko (Medizinprodukt Risikoklasse I oder IIa) wird die Bewertung durch das BfArM durchgeführt mit der Risikoklasse entsprechenden niedrigeren Evidenzanforderungen. Für DiGA mit höherem Risiko (Medizinprodukt Risikoklasse IIb oder III) wird für die Bewertung ein vollständiges HTA durch den G‑BA und das IQWiG für die Evidenzbeurteilung durchgeführt. Derzeit sind 15 DiGA im Verzeichnis gelistet (Stand 18.06.2021).

Allen Herangehensweisen der beschriebenen Länder gemein ist, dass als erster Schritt die Zuständigkeit von einer verantwortlichen Institution übernommen und eine öffentlich zugängliche Plattform für verfügbare DiGA geschaffen wird. Die dort registrierten DiGA werden zunächst auf ihre CE-Zertifizierung als Medizinprodukt und auf datenschutzrechtliche Aspekte überprüft. In einem weiteren Schritt wird die Kompatibilität mit nationalen Standards der eHealth-Strategien analysiert. In Ländern mit einer Wahlfreiheit unter gesetzlichen Krankenkassen (Niederlande, Deutschland) werden Versicherte auch über Zusatzangebote wie DiGA umworben. In Ländern ohne diese Wahlfreiheit (Belgien, Frankreich) ist der Zugang zu einer Refundierung deutlich strikter geregelt. Große Unterschiede zeigen sich, ob Länder einen Evidenznachweis vor der Refundierungsentscheidung einfordern (Belgien) oder ob sie Evidenz generierende Refundundierungsmodelle akzeptieren oder gar mitfinanzieren (Niederlande, Deutschland).

## Diskussion

Es liegt eine Vielzahl an Bewertungsinstrumenten für digitale Gesundheitsanwendungen (DiGA) vor. Nach einer detaillierten Analyse verschiedener Bewertungsinstrumente konnte eine große Heterogenität der Bewertungsinstrumente festgestellt werden. Um bei der Vielzahl an digitalen Gesundheitsanwendungen ein effektives, zeitsparendes Assessment zu ermöglichen und andererseits die Limitationen der jeweiligen Bewertungsinstrumente zu berücksichtigen, wird als Orientierungshilfe für Entscheidungsträger*innen eine Kombination verschiedener Bewertungsinstrumente in einem abgestuften Vorgehen empfohlen.

Ein systematisches Review zu Bewertungsinstrumenten für eHealth-Anwendungen von Vis et al. [7] identifizierte und analysierte 21 Bewertungsinstrumente. Als mögliches Bewertungsmodell für eHealth-Anwendungen wurde von Vis et al. eine Kombination des TEMPEST Frameworks [17], des CHEATS Frameworks [15], des UVON Frameworks [20] und des MAST Frameworks [16] genannt. Ebenso wird das NICE Evidence Standards Framework [10] von den Autor*innen empfohlen. Der Scope des systematischen Reviews von Vis et al. [7] lag jedoch bei eHealth-Anwendungen und berücksichtigte nur teilweise die Entwicklungen im Bereich mobiler digitaler Gesundheitsanwendungen („mHealth“).

Die in dieser Arbeit analysierten Bewertungsinstrumente können für mHealth-Anwendungen eingesetzt werden. In der Analyse zeigte sich insbesondere in Bezug auf eine Einteilung in Risikoklassen sowie auf technologiespezifische Aspekte wie Datenschutz und künstliche Intelligenz eine große Heterogenität. Bisher bietet nur das NICE Evidence Standards Framework [10] eine genaue Einteilung in definierte Risikoklassen mit entsprechend geforderten Studiendesigns, es stößt bei digitalen Gesundheitsanwendungen mit adaptiven Algorithmen jedoch an Grenzen. Die Herangehensweise für die Regulierung nach Risikoklassen von DiGA wurde bereits 2016 durch das IGES-Institut empfohlen [4]. Das Digi-HTA legt einen Fokus auf Aspekte der künstlichen Intelligenz, klammert jedoch rechtliche, soziale und ethische Aspekte aus. Insgesamt bestehen für die jeweils analysierten Bewertungsinstrumente Limitationen.

Die größte internationale Aufmerksamkeit bekommt das NICE Evidence Standards Framework [10]. So empfiehlt das IQWiG in der Befragung des INAHTA die Anwendung des NICE Evidence Standards Frameworks. Zudem wurde in einer Kolumne des Netzwerks evidenzbasierter Medizin eine Visualisierung der NICE Evidence Standards Framework-Anforderungen an neue DiGA erstellt [25]. Der hohe internationale Zuspruch für das NICE Evidence Standards Framework ergibt sich durch dessen praktische Anwendbarkeit: Mithilfe dieses Bewertungsinstruments wird eine DiGA zunächst einer funktionellen Klassifizierung nach Evidenzstufen unterzogen. DiGA, die trotz hoher Klassifizierung der Evidenzstufe 3b keinerlei Studien mit Komparator (bestenfalls randomisiert kontrollierte Studien) vorweisen können, können mithilfe dieses Bewertungsinstruments eindeutig von einer Refundierung ausgeschlossen werden.

In der Analyse ausgewählter Länder zeigt sich, dass Deutschland der gegebenen Situation (unzählige verfügbare Apps, geringe Evidenz zu Nutzenbelege) Rechnung trägt und nicht nur eine Registrierung möglicher verschreibbarer mHealth-Applikationen regelt, sondern darüber hinaus die Apps nach Risikoklassen – orientiert an der Medizinprodukteverordnung – kategorisiert und für höhere Risikoklassen (IIb und III) entsprechende Nutzenbelege durch Studien vorsieht. Diese können in Erprobungsstudien – nach Zulassung – erbracht werden. Deutschland ist mit dieser sehr klaren Vorgangsweise und der Einräumung der Möglichkeit prospektiver Evaluationsstudien (neben England) in Europa am weitesten fortgeschritten.

**Figure 1:**
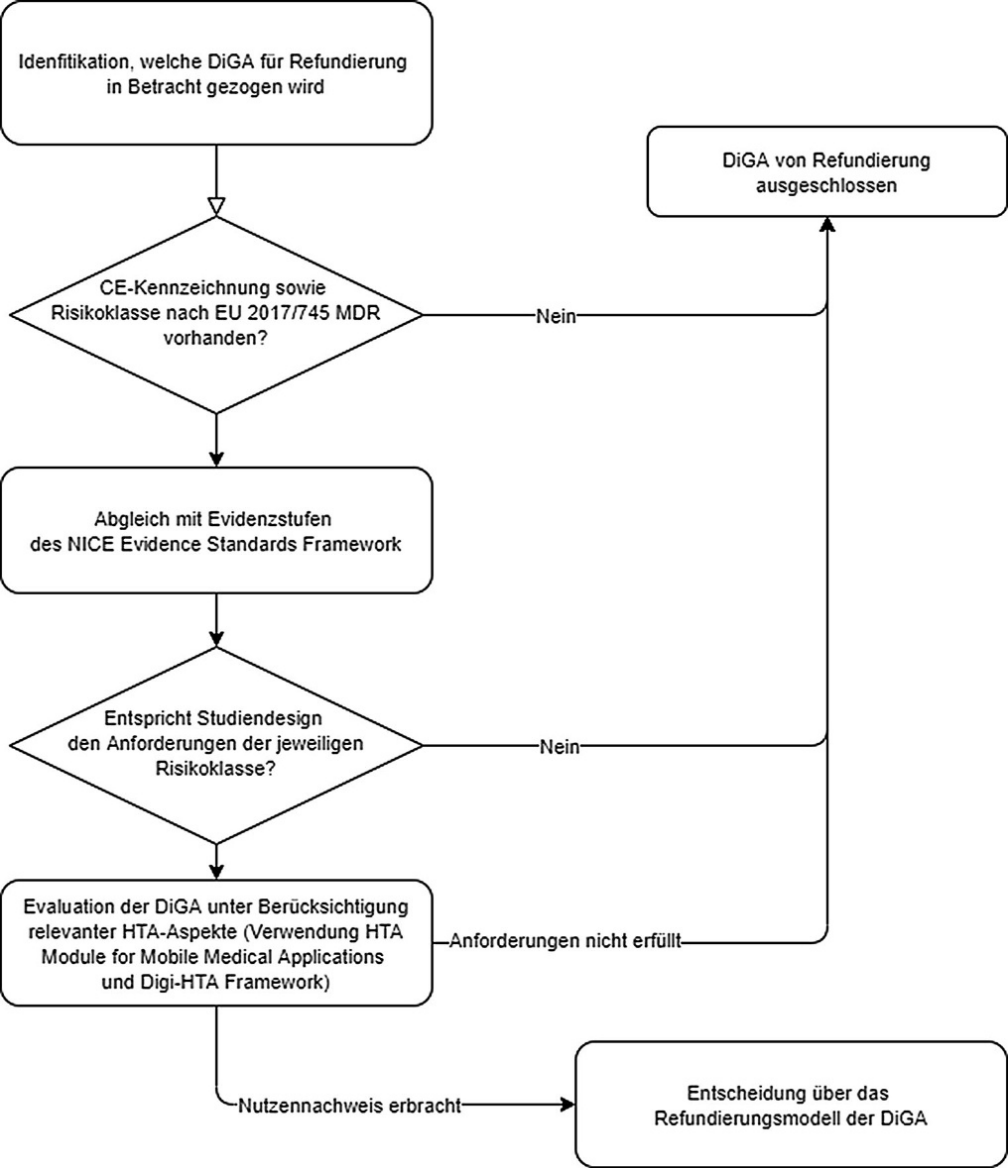

Eine gute Anwendbarkeit eines Bewertungsinstruments setzt eine bestmögliche Grundlage zur Entscheidungsfindung bei Refundierungsentscheidungen voraus. Auf der anderen Seite bietet ein Bewertungsinstrument auch Herstellern der Technologien eine wertvolle Orientierungshilfe für die Art von Evidenz, die für die jeweilige DiGA vorzulegen ist. Auch wenn sich DiGA von konventionellen Interventionen im Gesundheitswesen unterscheiden, sollten sie dabei dieselben Evidenzanforderungen zu Nutzen, möglichen Schäden und entstehenden Kosten erfüllen. Für evidenzbasierte Refundierungsentscheidungen einzelner digitaler Gesundheitsanwendungen bedarf es Mindestvoraussetzungen für gewünschte Studiendesigns. Nur wenige der identifizierten Bewertungsinstrumente bieten hier eine solide Orientierungshilfe. Eine Möglichkeit ist ein abgestufter Bewertungsprozess, wie er in Abb. 1 dargestellt ist.

Für Österreich wird vorgeschlagen, sich an den ersten Erfahrungen aus anderen Ländern zu orientieren [6]: zunächst zu überprüfen, ob eine CE-Kennzeichnung vorliegt und nach welcher Risikoklasse nach EU-Verordnung 2017/745 über Medizinprodukte [2] die DiGA klassifiziert wurde. Anschließend ist ein Abgleich der Risikoklasse nach EU-Verordnung 2017/745 mit den Evidenzerfordernissen der Evidenzstufen des NICE Evidence Standards Frameworks [10] zu empfehlen. In einem nächsten Schritt sollten ein Screening und Scoping der vorhandenen Evidenz erfolgen. Randomisierte, kontrollierte Studien, die die DiGA mit dem derzeitigen Versorgungsstandard vergleichen, stellen das höchste Maß an Evidenz dar. DiGA ohne entsprechend vorliegende Studien sollten (vorerst) von einer Refundierung ausgeschlossen werden, bis Evidenz vorgelegt wird. Für DiGA, welche die Anforderungen erfüllen, können dann die relevanten HTA-Aspekte definiert, die sich an den Erwartungen der Krankenkassen und Versprechungen der Hersteller orientieren, und infolge bewertet werden, um eine fundierte Entscheidung zum nachgewiesenen Nutzen und den erwartbaren Effekten der digitalen Gesundheitsanwendung treffen zu können. Zuletzt wird empfohlen, diesen abgestuften Prozess zu pilotieren und ggf. zu adaptieren.
